# Electrostatic theory of rectangular waveguides filled with anisotropic media

**DOI:** 10.1038/s41598-021-04293-6

**Published:** 2021-12-31

**Authors:** Afshin Moradi

**Affiliations:** Department of Engineering Physics, Kermanshah University of Technology, Kermanshah, Iran

**Keywords:** Microwave photonics, Metamaterials

## Abstract

The electrostatic (or, in a better word, quasi-electrostatic) theory of waves propagation in a long, rectangular waveguide having perfect electric conductor walls that filled with an anisotropic medium (here, a medium of nanowire-based hyperbolic metamaterials) is presented. Some data on characteristics of these waves are prepared. The presented results include electrostatic field configurations (modes) that can be supported by such structures and their corresponding cutoff frequencies, group velocities, power flows and storage energies.

## Introduction

A rectangular waveguide is a long hollow tube (compared with its cross section) of rectangular cross section with four perfect electric conductor (PEC) walls. Investigation of electromagnetic properties of waveguides with PEC boundaries and filled with various anisotropic media, based on full Maxwell’s equations, has been receiving considerable interests for decades^1–7^. For instance, Van Trier^1^ studied how the well-known electromagnetic modes in a waveguide are modified in the presence of a magnetized plasma into the waveguide. Also, the field configurations (modes) in hollow (rectangular/circular) waveguides and cylindrical dielectric waveguides were presented in Refs.^8,9^, respectively. Furthermore, there are many interesting works in (rectangular/circular) waveguides filled with metamaterials^10–16^. For instance, Xu^10^ studied the characteristics of rectangular waveguides filled with anisotropic metamaterials and derived a general condition of the existence of TE and TM modes in these waveguides. In particular, Bhardwaj et al.^15^ investigated the propagation of electromagnetic waves in a cylindrical waveguide filled with hyperbolic metamaterials (HMMs). They found that such a waveguide has no cutoff frequency for propagating TM and TE modes.

The HMMs^17–21^, which form a subclass of metamaterials, has attracted the attention of many authors. More recently, we have used the electrostatic theory to study the propagation of electrostatic waves in unbounded^22^ and bounded^23–25^ media of nanowire-based HMMs. A HMM exhibits a unique property of having anisotropy with simultaneously different signs of the permittivity tensor components, i.e, $\varepsilon _{\bot }$ and $\varepsilon _{\Vert }$^19,20^. In general, two types of HMMs can be considered. In type I, $\varepsilon _{\bot }$ is positive and $\varepsilon _{\Vert }$ is negative and then the HMM medium is called dielectric. In type II, $\varepsilon _{\bot }$ is negative and $\varepsilon _{\Vert }$ is positive, and HMM medium is called a metallic medium.

But, *can quasi-electrostatic waves, or more commonly, electrostatic waves propagate in a waveguide with PEC walls*? Actually, a hollow waveguide cannot support electrostatic modes, but when it is filled with an anisotropic medium the existence of electrostatic waves may be possible. However, despite the relatively extensive investigations on electromagnetic characteristics of waveguides, to the best of our knowledge, the electrostatic wave propagation inside the rectangular waveguides filled with anisotropic media are less well-known in the theory of waveguides. For this reason, standard text and reference books present only the modal electromagnetic field distribution of an electromagnetic hollow waveguide^26^.

In the present work, we present the electrostatic theory of waves propagation in a long, rectangular waveguide containing an anisotropic medium, here a medium of nanowire-based HMMs. The results derived by employing electrostatic theory, indicates the velocity of light in free space must be much more than phase velocity^27^. Actually, the frequency term in Helmholtz equation is dropped here and the modified Laplace equation is solved instead.

## Spectroscopy of electrostatic modes of a planar slab of nanowire-based HMMs

**Figure 1:**
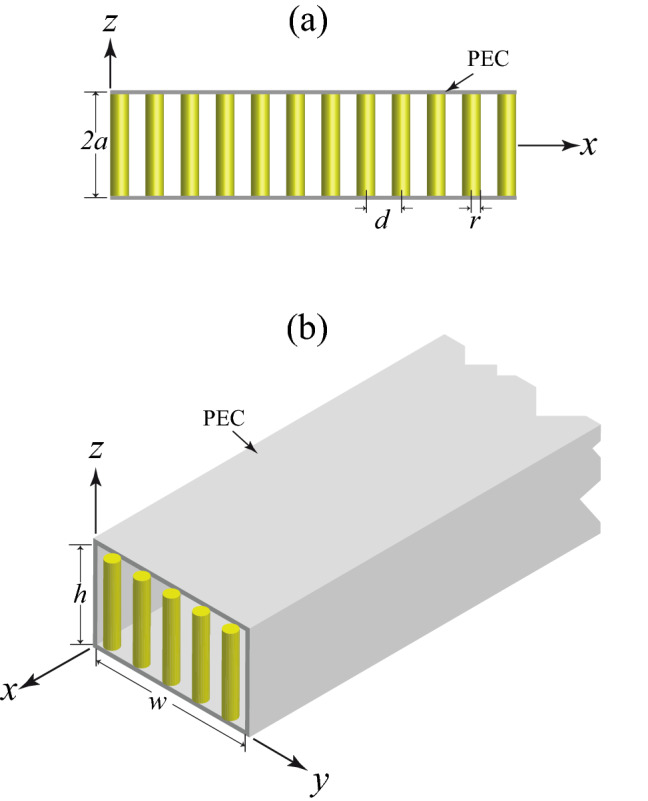
(**a**) Side view of a planar slab of nanowire-based HMMs. The array of nanowires has areal density *N* with axes parallel to *z*-axis. Also, *r* is the radius of a nanowire and $d=1/\sqrt{N}$ is the separation between the nanowires. The PEC interfaces separating the slab ($-a\le z\le a$). (**b**) Isometric view of a rectangular waveguide filled with a medium of nanowire-based HMMs with its appropriate dimensions and PEC boundaries.

One type of electrostatic waveguide is a planar slab of an anisotropic medium (here, a medium of nanowire-based HMMs in the perpendicular configuration^25^) of height 2*a* with two PEC boundaries, as shown in panel (a) of Fig. 1. Note that in the perpendicular configuration the PEC boundaries are perpendicular to the axes of nanowires. The parallel configuration is another configuration, where the axes of nanowires are parallel to the PEC boundaries. The array of nanowires has areal density *N* with axes parallel to *z*-axis. Also, *r* and $d=1/\sqrt{N}$ are the radius of a nanowire and the separation between the nanowires, respectively. To simplify the analysis of the structure, in this section we reduce the problem to a 2D one (its length in the *y* direction is infinite) so that $\partial /\partial y=0$. Although in practice the dimensions of the structure are finite, the 2D approximation not only simplifies the structure but also sheds insight into the characteristics of the structure. In general case, the cross section of the slab is a rectangular with finite height and width.

We now investigate the behaviors of electrostatic bulk modes of a planar slab of nanowire-based HMMs, based on the field analysis. Let us assume that the waves are traveling in the *x* direction, and the structure is infinite in that direction as illustrated in panel (a) of Fig. 1. We shall obtain the general expression for dispersion relation of the electrostatic waves. For the present problem, the relative dielectric tensor of the anisotropic medium is

1 $$\begin{aligned} {\underline{\varepsilon }}(\omega )=\left( \begin{array}{lll} \varepsilon _{\bot } &{} 0 &{} 0\\ 0 &{} \varepsilon _{\bot } &{} 0\\ 0 &{} 0 &{} \varepsilon _{\Vert }\\ \end{array} \right) \;, \end{aligned}$$

where $\varepsilon _{\bot }$ and $\varepsilon _{\Vert }$ that are the effective transversal and longitudinal permittivities, respectively, may be expressed by^19^

2 $$\begin{aligned}&\varepsilon _{\bot }=\varepsilon _{\text {d}}\dfrac{(1+f) \varepsilon _{\text {m}}+(1-f)\varepsilon _{\text {d}}}{(1-f) \varepsilon _{\text {m}}+(1+f)\varepsilon _{\text {d}}}, \end{aligned}$$

3 $$\begin{aligned}&\varepsilon _{\Vert }=f\varepsilon _{\text {m}}+(1-f) \varepsilon _{\text {d}}, \end{aligned}$$

where $\varepsilon _{\text {m}}=\varepsilon _{\infty } -\omega _{\text {p}}^{2}/\omega \left( \omega +i\gamma \right)$ shows the relative permittivity of a nanowire (for example a narrow-gap semiconductor nanowire such as InSb^27,28^), $\varepsilon _{\infty }$ is the high-frequency bulk permittivity, $\omega _{\text {p}}$ is the electron plasma angular frequency, $\gamma$ is the damping constant, $f=\pi r^{2}N$ is the filling factor of nanowires in the *xy* section of medium and $\varepsilon _{\text {d}}$ is the relative permittivity of insulator host.

Under the electrostatic approximation, the electric field can be represented by the gradient of an electric potential $\Phi$. The Maxwell’s equation $\nabla \cdot \mathbf{D }=0$, gives the wave equation for the electrostatic potential of an unbounded medium of nanowire-based HMMs

4 $$\begin{aligned} \left[ \varepsilon _{\bot }\dfrac{\partial ^{2}}{\partial x^{2}} +\varepsilon _{\Vert }\dfrac{\partial ^{2}}{\partial z^{2}} \right] \Phi =0, \end{aligned}$$

where all the field quantities are assumed to have the harmonic time dependence of the form $\exp (-i\omega t)$. Note that $\omega$ is angular frequency of an electrostatic wave in the system.

In deriving the electrostatic mode spectrum of a planar slab of nanowire-based HMMs, we use the following general separated-variable solution for traveling waves in the $+x$ direction

5 $$\begin{aligned} \Phi (x,z)=\left( A \sin \kappa z+B \cos \kappa z\right) \exp (ikx). \end{aligned}$$

Substituting this into Eq. (4), we get

6 $$\begin{aligned} \varepsilon _{\bot }k^{2} +\varepsilon _{\Vert }\kappa ^{2}=0. \end{aligned}$$

Equation (6) shows that a homogeneous bulk electrostatic plane wave corresponding to real *k* and $\kappa$ is possible only when $\varepsilon _{\bot }/\varepsilon _{\Vert }$ is negative. Field existing within this waveguide must be characterized by zero tangential components of electric field at the PEC walls. However, the boundary conditions at the two PEC walls can be written as

7 $$\begin{aligned} \Phi \vert _{z=-a,a}=0. \end{aligned}$$

Now, by applying the mentioned boundary conditions we can find the relationship between the constants *A* and *B*, as

8 $$\begin{aligned} \pm A\sin \kappa a + B \cos \kappa a=0. \end{aligned}$$

To satisfy Eq. (8) we consider separately two categories: even and odd modes. For an even mode $A=0$ and $\kappa a=\left( n+1/2\right) \pi$ with $n=0,1,2,\ldots$ Therefore $\kappa a=\left( n+1/2\right) \pi$ is the dispersion relation for electrostatic waves with symmetric potential functions. For an odd mode $B=0$ and we have $\kappa a=n\pi$ that is the dispersion relation for electrostatic waves with anti-symmetric potential functions. The even and odd mode dispersion relations can be combined into a single equation. The result is

9 $$\begin{aligned} \kappa a=n\dfrac{\pi }{2}, \end{aligned}$$

where this equation corresponds to an even mode for *n* odd, and to an odd mode for *n* even. In the special case, for $\varepsilon _{\infty }=1=\varepsilon _{\text {d}}$ and $\gamma =0$, from Eq. (9), we find

10 $$\begin{aligned} \omega _{\pm }=\dfrac{1}{2}\left\{ 1+f\pm \dfrac{\left[ \left( 1-3f\right) ^{2} \left( \dfrac{n \pi }{2ka} \right) ^{2}+\left( 1+f \right) ^{2} \right] ^{1/2}}{\left[ 1+\left( \dfrac{n \pi }{2ka} \right) ^{2} \right] ^{1/2}} \right\} ^{1/2}. \end{aligned}$$

If $k\rightarrow 0$, then $\omega _{+}\rightarrow \sqrt{1-f}\omega _{\text {p}}/\sqrt{2}$ and $\omega _{-}\rightarrow \sqrt{f}\omega _{\text {p}}$. If $k\rightarrow \infty$, then $\omega _{+}\rightarrow \sqrt{1+f}\omega _{\text {p}}/\sqrt{2}$ and $\omega _{-}\rightarrow 0$. This means that this system represents a bulk electrostatic band filter with the frequency bands $\sqrt{1-f}/\sqrt{2}\le \omega _{+}/\omega _{\text {p}}\le \sqrt{1+f}/\sqrt{2}$ and $0< \omega _{-}/\omega _{\text {p}}\le \sqrt{f}$. Note that the mentioned effective medium approximation that we use in the present study is valid when the radius of a nanowire is a lot smaller than the wavelength of the waves under consideration. Also the nanowires must be well separated. In particular, the conductor walls must behave as PEC walls and thus they must have constant potential. Therefore, the angular frequency is assumed to be restricted to the range $0<\omega _{-}/\omega _{\text {p}}\le \sqrt{f}$ with the consequence that we can choose the appropriate frequency of plane electrostatic wave in the microwave regime and appropriate radius of a nanowire in the present HMM medium. For instance, for InSb wires the electron plasma frequency $\omega _{\text {p}}/2\pi =4.9\;$THz^27,28^ is approximately $61\;\upmu$m in wavelength. If one uses the angular frequency region of $\omega _{-}\approx \sqrt{f}\omega _{\text {p}}$, the wavelength under consideration is $61/\sqrt{f}\;\upmu$m. Thus, for $r=5\;$nm and $d=1\;\upmu$m, we find $\sqrt{f}\approx 8.8\times 10^{-3}$ and therefore $\lambda \gg r,d$ which indicates the mentioned effective medium approximation is valid in the present study. Furthermore, we find $\omega _{-}/2\pi \le 43.4\;$GHz and therefore one may conclude the condition that conductor walls must behave as PEC walls is satisfied. Note that for the angular frequency range $0<\omega _{-}/\omega _{\text {p}}\le \sqrt{f}$, one can find that $\varepsilon _{\bot }$ is positive and $\varepsilon _{\Vert }$ is negative, thus the present system for our purpose is a HMM of type I.

**Figure 2:**
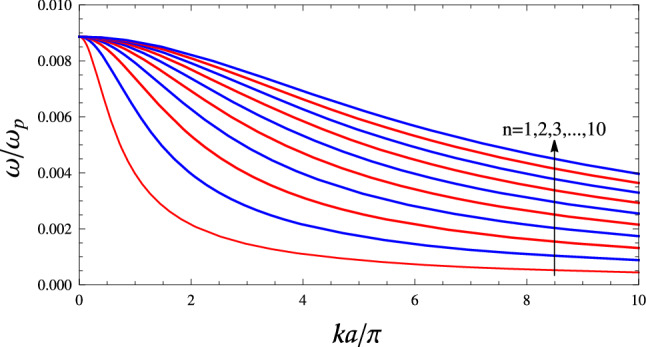
Dispersion curves of the first ten bulk electrostatic modes of a planar slab of nanowire-based HMMs with $r=5\;$nm, $d=1\;\upmu$m and PEC boundaries, using Eq. (10). The modes $n=1,3,5,7,9$ are even (symmetric) modes and $n=2,4,6,8$ are odd (anti-symmetric) modes. The slope of curves is negative in the frequency range $0<\omega _{-}\le \sqrt{f}\omega _{\text {p}}$ for all mode orders. Therefore, these modes called backward waves. For a backward wave, the directions of group velocity (power flow) and phase velocity (phase propagation) are mutually opposite.

The dispersion curves of modes of a planar slab of nanowire-based HMMs with $r=5\;$nm, $d=1\;\upmu$m for various values of *n*, using Eq. (10) are depicted in Fig. 2. An infinite number of bulk electrostatic waves with different values of *n* can exist in the slab, which depend on the conditions of excitation. It is clear from Fig. 2 that the frequency of an electrostatic mode decreases monotonically with wavenumber throughout the allowed frequency range $0<\omega _{-}\le \sqrt{f}\omega _{\text {p}}$. Therefore, these electrostatic modes are backward waves. Furthermore, all backward modes have equal cutoff frequency $\omega _{-}=\sqrt{f}\omega _{\text {p}}$. Hence, there is no frequency band where only a single mode propagates. Note that using the simple formula $v_{\text {g}}=d\omega /dk$ the group velocity of bulk modes of the system can be simply computed in an analytical way.

## Spectroscopy of electrostatic modes of a rectangular waveguides filled with a medium of nanowire-based HMMs

Now, consider a long, rectangular waveguide of nanowire-based HMM having PEC walls of dimensions *w* and *h* in the *y* and *z* directions respectively. Geometry of the problem is presented in panel (b) of Fig. 1. It is our purpose to determine the various electrostatic modes that can exist inside this rectangular electrostatic waveguide.

In this case, the wave equation for the electrostatic potential inside the HMM medium can be written as

11 $$\begin{aligned} \left[ \varepsilon _{\bot }\left( \dfrac{\partial ^{2}}{\partial x^{2}}+\dfrac{\partial ^{2}}{\partial y^{2}}\right) +\varepsilon _{\Vert }\dfrac{\partial ^{2}}{\partial z^{2}} \right] \Phi =0. \end{aligned}$$

Again, the solution to Eq. (11) can be obtained by using the separation of variables method. In general, the solution to $\Phi (x,y,z)$ for traveling electrostatic waves in the $+x$ direction can be written as

12 $$\begin{aligned} \Phi (x,y,z)=\left( C \sin \alpha y+D \cos \alpha y\right) \left( E \sin \beta z+F \cos \beta z\right) \exp (ikx). \end{aligned}$$

Substituting this equation into Eq. (11), we get

13 $$\begin{aligned} \varepsilon _{\bot }\left( k^{2}+\alpha ^{2}\right) +\varepsilon _{\Vert }\beta ^{2}=0. \end{aligned}$$

Note that *C*, *D*, *E*, and *F* are constants that will be evaluated by the appropriate boundary conditions on the walls of the waveguide. Here, the boundary conditions at the four PEC walls are

14 $$\begin{aligned}&\Phi \vert _{y=0,w}=0. \end{aligned}$$

15 $$\begin{aligned}&\Phi \vert _{z=0,h}=0. \end{aligned}$$

To satisfy the above boundary conditions for electrostatic modes, we choose

16 $$\begin{aligned} \alpha= & {} \dfrac{m \pi }{w},\quad D=0, \end{aligned}$$

17 $$\begin{aligned} \beta= & {} \dfrac{n \pi }{h},\quad F=0, \end{aligned}$$

where *m* and *n* are integers. Only the trivial solution $\Phi =0$ is possible when $m=0\; or/and\; n=0$. The electric potential and electric field components are

18 $$\Phi (x,y,z) = A_{{mn}} \sin \left( {\frac{{m\pi y}}{w}} \right)\sin \left( {\frac{{n\pi z}}{h}} \right)\exp (ikx),$$

19 $$E_{x} (x,y,z) = - ikA_{{mn}} \sin \left( {\frac{{m\pi y}}{w}} \right)\sin \left( {\frac{{n\pi z}}{h}} \right)\exp (ikx),$$

20 $$E_{y} (x,y,z) = - A_{{mn}} \left( {\frac{{m\pi }}{w}} \right)\cos \left( {\frac{{m\pi y}}{w}} \right)\sin \left( {\frac{{n\pi z}}{h}} \right)\exp (ikx),$$

21 $$E_{z} (x,y,z) = - A_{{mn}} \left( {\frac{{n\pi }}{h}} \right)\sin \left( {\frac{{m\pi y}}{w}} \right)\cos \left( {\frac{{n\pi z}}{h}} \right)\exp (ikx).$$

The dispersion equation for the modes can be written as

22 $$\begin{aligned} k =\dfrac{1}{\sqrt{\varepsilon _{\bot }}} \left[ -\varepsilon _{\bot }\left( \dfrac{m\pi }{w}\right) ^{2} -\varepsilon _{\Vert }\left( \dfrac{n\pi }{h}\right) ^{2}\right] ^{1/2}. \end{aligned}$$

**Figure 3:**
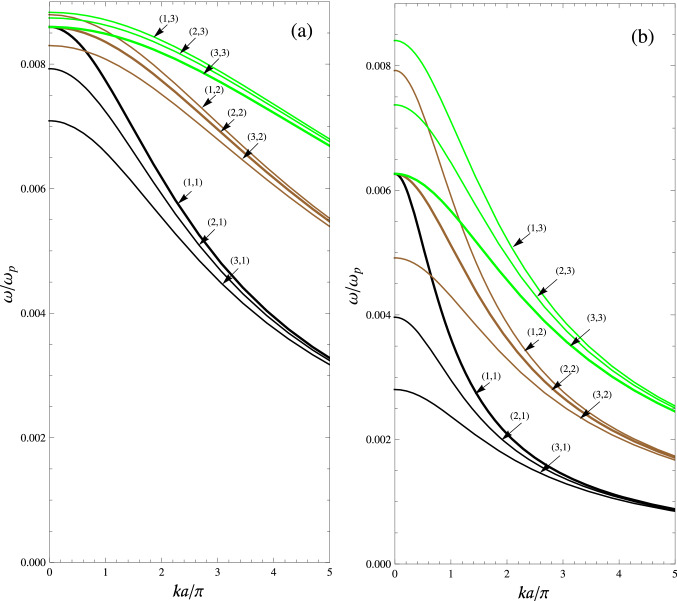
Dispersion curves of electrostatic modes (*m*, *n*) of a waveguide filled with a medium of nanowire-based HMMs with $r=5\;$nm, $d=1\;\upmu$m and PEC boundaries, for $n=1,2,3$, and $m=1,2,3$. (**a**) A rectangular waveguide ($2h=2a=w$), using Eq. (23). (**b**) A square waveguide ($h=2a=w$), using Eq. (24).

**Figure 4:**
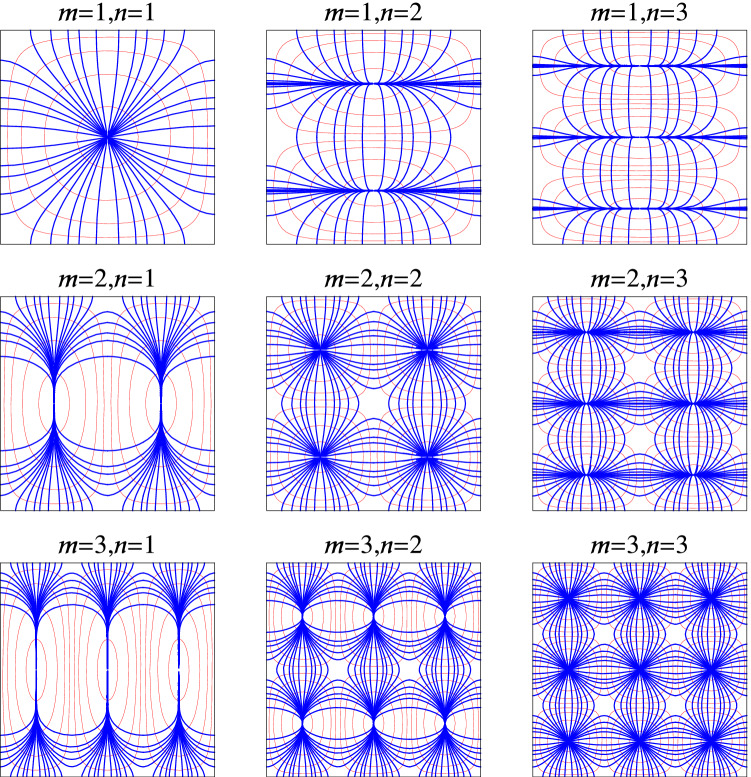
Electric field patterns (blue lines) and their corresponding cross sections of equipotential surfaces (red lines) for the first nine electrostatic modes of a square waveguide ($h=w$) with PEC boundaries and filled with an anisotropic medium, for example a medium of nanowire-based HMMs.

**Figure 5:**
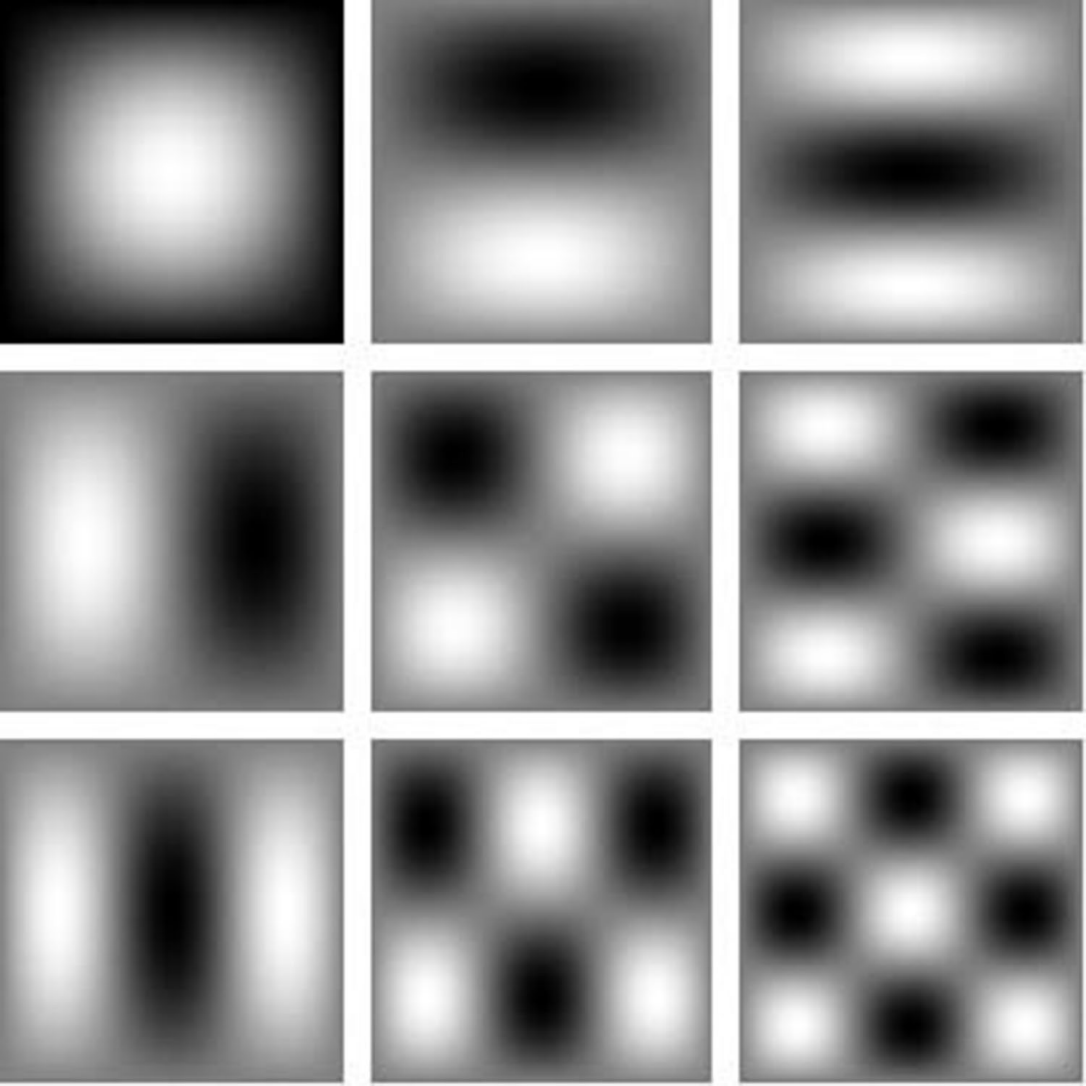
Cross section of bulk charge density of a square waveguide ($h=w$) with PEC boundaries and filled with an anisotropic medium (for example a medium of nanowire-based HMMs) corresponding to the Fig. 4. The white color corresponds to positive charge and black color to negative charge. The first left column (from top to bottom) shows modes (1, 1), (2, 1) and (3, 1), respectively, the second column shows modes (1, 2), (2, 2) and (3, 2), respectively and the third column shows modes (1, 3), (2, 3) and (3, 3), respectively.

Since there are infinite combinations of *m* and *n*, an infinite number of electrostatic modes can be found. In the special case, for $\varepsilon _{\infty }=1=\varepsilon _{\text {d}}$ and $\gamma =0$, from Eq. (22), we find

23 $$\begin{aligned} \omega _{\pm }=\dfrac{1}{2}\left\{ 1+f\pm \dfrac{ \left[ \left( 1-3f\right) ^{2} \left( \dfrac{nw}{h}\right) ^{2} +\Upsilon _{\text {r}} \left( 1+f \right) ^{2} \right] ^{1/2}}{\left[ \Upsilon _{\text {r}}+\left( \dfrac{nw}{h}\right) ^{2} \right] ^{1/2}} \right\} ^{1/2}, \end{aligned}$$

where $\Upsilon _{\text {r}}=m^{2}+\left( \dfrac{kw }{\pi } \right) ^{2}$. Again, we take the symbol “−” in Eq. (23) that is in agreement with our model throughout the allowed frequency range $0<\omega _{-}\le \sqrt{f}\omega _{\text {p}}$. Assuming $h=w=2a$, the dispersion relation of a square waveguide can be written as

24 $$\begin{aligned} \omega _{\pm }=\dfrac{1}{2}\left\{ 1 +f\pm \dfrac{\left[ \left( 1-3f\right) ^{2} n^{2} +\Upsilon _{\text {s}}\left( 1+f \right) ^{2} \right] ^{1/2}}{\left( \Upsilon _{\text {s}}+n^{2} \right) ^{1/2}} \right\} ^{1/2}, \end{aligned}$$

where $\Upsilon _{\text {s}}=m^{2}+\left( \dfrac{2ka }{\pi } \right) ^{2}$. The dispersion curves for electrostatic modes (*m*, *n*) ($n=1,2,3$ and $m=1,2,3$) of a waveguide filled with a medium of nanowire-based HMMs with $r = 5\;$nm, $d=1\;\mu$m are depicted in panels (a) and (b) of Fig. 3. Panel (a) shows the results for a rectangular waveguide ($2h = 2a = w$), using Eq. (23) and panel (b) shows the results for a square waveguide ($h = 2a = w$), using Eq. (24). Furthermore, the values of normalized cutoff frequencies for a rectangular waveguide ($w = 2h = 2a$), using Eq. (23) and a square waveguide ($w = h = 2a$) using Eq. (24) are listed in Table 1. We see that mode (3, 1) is the dominate mode for electrostatic modes (*m*, *n*) (with $n=1,2,3$ and $m=1,2,3$). Also, modes (1, 1), (2, 2), and (3, 3) have equal cutoff frequencies. In particular, the *yz* cross section electric field and their corresponding cross sections of equipotential surfaces for the first nine electrostatic modes of a square waveguide are plotted in Fig. 4. Field configurations of a rectangular waveguide are very similar with Fig. 4 (not shown here). By comparing the results in Fig. 4 of^8^ with Fig. 4 in the present work, one can conclude that the electric field pattern of electrostatic mode (*m*, *n*) is similar with electric field pattern of ${\text {TM}}_{{mn}}$ modes for a hollow waveguide. Furthermore, in Fig. 5 we show the *yz* cross section of bulk charge density of a square waveguide corresponding to the Fig. 4. The white color corresponds to positive charge and black color to negative charge.

**Table 1:** Normalized cutoff frequencies for electrostatic modes (*m*, *n*) ($n=1,2,3$ and $m=1,2,3$) of a waveguide filled with a medium of nanowire-based HMMs with $r=5\,$nm, $d=1\,\upmu$m, using Eqs. (23) and (24).

(*m*, *n*)	$$\begin{array}{ll} \omega _{-}/\omega _{\text {p}}\\ (w=2h=2a)\\ \end{array}$$	$$\begin{array}{ll} \omega _{-}/\omega _{\text {p}}\\ (w=h=2a)\end{array}$$
(3, 1)	$7.0\times 10^{-3}$	$2.8\times 10^{-3}$
(2, 1)	$7.9\times 10^{-3}$	$3.9\times 10^{-3}$
(3, 2)	$8.2\times 10^{-3}$	$4.9\times 10^{-3}$
$$\begin{array}{ll} (1,1)\\ (2,2)\\ (3,3)\\ \end{array}$$	$8.5\times 10^{-3}$	$6.2\times 10^{-3}$
(2, 3)	$8.7\times 10^{-3}$	$7.4\times 10^{-3}$
(1, 3)	$8.8\times 10^{-3}$	$8.4\times 10^{-3}$

The electrostatic waves that are created and propagating inside the present waveguide have power associated with them. To find the power flowing down the waveguide, it is first necessary to find the cycle-averaged of power density directed along the axis of the waveguide. The power flowing along the waveguide can then be obtained by integrating the axial directed power density over the cross section of the waveguide.

For the waveguide geometry of Fig. 1, the *x*-directed power density can be written as^29^ 

25 $$\begin{aligned} S_{x}=-\dfrac{\varepsilon _{0}}{2}\text{Re} \left[ \varepsilon _{\bot }\Phi \dfrac{\partial }{\partial t}\dfrac{\partial }{\partial x}\Phi ^{*}\right] , \end{aligned}$$

in the complex-number representation, where $^{*}$ denotes complex conjugation, and Re denotes taking the real part. Use of Eq. (18) into Eq. (25), allows the *x*-directed power density of Eq. (25) for the electrostatic modes can be written as

26 $$\begin{aligned} S_{x}=-\dfrac{\varepsilon _{0}\omega k}{2} \varepsilon _{\bot } \vert A_{mn}\vert ^2 \sin ^{2} \left( \dfrac{m \pi y}{w}\right) \sin ^{2} \left( \dfrac{n \pi z}{h}\right) . \end{aligned}$$

Note that $\varepsilon _{\bot }$ and $\varepsilon _{\Vert }$ are real since we neglected damping in the present study. The associated power is obtained by integrating Eq. (26) over the cross section ${\mathcal {A}}=hw$ of the guide, as

27 $$\begin{aligned} P_{mn} =\int _{0}^{h}\int _{0}^{w} S_{x} dydz=-\dfrac{\varepsilon _{0}\omega k}{8} \varepsilon _{\bot }hw \vert A_{mn}\vert ^2. \end{aligned}$$

Also, the cycle-averaged of energy density distribution associated with the waves can be written as^29^ 

28 $$\begin{aligned} u=\dfrac{\varepsilon _{0}}{4} \nabla \Phi ^{*}\cdot \left[ \dfrac{d \left( \omega {\underline{\varepsilon }}\right) }{d\omega }\cdot \nabla \Phi \right] . \end{aligned}$$

After substitution Eq. (18) into Eq. (28), we obtain

29 $$\begin{aligned} u&=\dfrac{\varepsilon _{0}k^{2}}{4} \dfrac{d \left( \omega \varepsilon _{\bot }\right) }{d\omega } \vert A_{mn}\vert ^2 \sin ^2 \left( \dfrac{m \pi y}{w}\right) \sin ^2 \left( \dfrac{n \pi z}{h}\right) \nonumber \\&\quad +\dfrac{\varepsilon _{0}}{4}\left( \dfrac{m \pi }{w}\right) ^2\dfrac{d \left( \omega \varepsilon _{\bot }\right) }{d\omega } \vert A_{mn}\vert ^2 \cos ^2 \left( \dfrac{m \pi y}{w}\right) \sin ^2 \left( \dfrac{n \pi z}{h}\right) \nonumber \\&\quad +\dfrac{\varepsilon _{0}}{4}\left( \dfrac{n \pi }{h}\right) ^2\dfrac{d \left( \omega \varepsilon _{\Vert }\right) }{d\omega } \vert A_{mn}\vert ^2 \sin ^2 \left( \dfrac{m \pi y}{w}\right) \cos ^2 \left( \dfrac{n \pi z}{h}\right) . \end{aligned}$$

The associated storage energy is obtained by integrating Eq. (29) over the cross section ${\mathcal {A}}$ of the waveguide, as

30 $$\begin{aligned} U_{mn}=\dfrac{\varepsilon _{0}}{16}wh\omega \vert A_{mn}\vert ^2 \left\{ \left[ k^{2}+\left( \dfrac{m \pi }{w}\right) ^2\right] \dfrac{d \varepsilon _{\bot } }{d\omega } +\left( \dfrac{n \pi }{h}\right) ^2\dfrac{d \varepsilon _{\Vert } }{d\omega }\right\} . \end{aligned}$$

As mentioned before, using the simple formula $v_{\text {g}}=d\omega /dk$ and Eq. (22), the group velocity of electrostatic modes of the system can be simply computed. However, in the absence of the damping effects, the group velocity of the waves is also equal with the ratio of the power flow to the storage energy, such as

31 $$\begin{aligned} v_{\text {g}}=\dfrac{P_{mn} }{U_{mn} }=\dfrac{-2 k \varepsilon _{\bot }}{ \left[ k^{2}+\left( \dfrac{m \pi }{w}\right) ^2\right] \dfrac{d \varepsilon _{\bot } }{d\omega } +\left( \dfrac{n \pi }{h}\right) ^2\dfrac{d \varepsilon _{\Vert } }{d\omega }}. \end{aligned}$$

Since all investigations in this work is based on electrostatic theory, the equality of above formula with the result obtained using $v_{\text {g}}=d\omega /dk$ is a manifestation of self-consistency and general validity of presented results in the electrostatic theory.

## Conclusion

We have presented the theory of electrostatic bulk waves propagation in a long, rectangular PEC waveguide containing an anisotropic medium (here, a medium of nanowire-based HMMs). We have derived general expression for dispersion equation of the waves and then presented the plots the electrostatic field distributions in such electrostatic waveguides. The results show that the electric field pattern of electrostatic modes are similar with electric field pattern of ${\text {TM}}_{{mn}}$ modes in the rectangular hollow waveguides^8^. Also, we have found that all modes with $m=n$ have equal cutoff frequencies and mode $(m=3,n=1)$ is the dominant mode for electrostatic modes (*m*, *n*) (with $n=1,2,3$ and $m=1,2,3$) in the electrostatic waveguides. However, in general the dominate mode of the system cannot be found. Finally, we have verified the obtained results by showing that group velocity of the waves is the same as energy velocity (i.e, the ratio of the power flow to the storage energy). Because of the possibility of electrostatic waves propagation in a rectangular waveguides filled with anisotropic media, they may be used in the development of new waveguides using guided electrostatic waves.

## Data Availability

The data that supports the findings of this study are available within the article.
